# Struggling to cope in an unknown realm: a qualitative study of moral resilience in frontline healthcare professionals in Swedish hospital wards during a pandemic crisis

**DOI:** 10.1136/bmjopen-2025-104065

**Published:** 2026-03-23

**Authors:** Lena Nordgren, Malin Lohela Karlsson, Emelie Condén Mellgren, Camilla Göras, Petronella Bjurling-Sjöberg

**Affiliations:** 1 Uppsala universitet Disciplinary Domain of Medicine and Pharmacy, Uppsala, Sweden; 2 Centre for Clinical Research, County Council in Sörmland, Eskilstuna, Sweden; 3 Department of Public Health and Caring Sciences, Uppsala Universitet, Uppsala, Sweden; 4 Centre for Clinical Research, Region Västmanland, Västmanland Hospital Västerås, Uppsala universitet Medicinska fakulteten, Västerås, Sweden; 5 Centre for Clinical Research, Uppsala Universitet, Västerås, Sweden; 6 Department of Caring Sciences, University of Gavle Department of Occupational Health and Psychology, Gavle, Sweden; 7 School of Health and Welfare, Department of Caring Sciences, Dalarna University, Falun, Sweden; 8 Public Health and Caring Sciences, Health Service Research, Uppsala University Faculty of Medicine, Uppsala, Sweden; 9 Department of Patient Safety, Region Sörmland, Eskilstuna, Sweden

**Keywords:** COVID-19, QUALITATIVE RESEARCH, MENTAL HEALTH

## Abstract

**Abstract:**

**Objectives:**

This study aimed to explore the process of moral resilience among frontline healthcare professionals. By delving into experiences of handling moral challenges during a pandemic crisis, we aimed to understand dimensions of moral resilience, affecting factors and consequences. This understanding can inform the implementation of interventions to support healthcare professionals’ well-being and ability to deliver high-quality care, under both routine and extreme conditions.

**Design:**

A qualitative exploratory study was conducted using grounded theory methodology. Data were collected retrospectively through written narratives and individual interviews (September to November 2020).

**Setting:**

General hospital wards allocated for patients with COVID-19 in two Swedish healthcare regions.

**Participants:**

46 informants, comprising registered nurses, nursing assistants, physicians, managers and allied health professionals.

**Results:**

A conceptual model is presented that describes and explains the process of moral resilience among frontline healthcare professionals working in general hospital wards during a pandemic crisis. The model reveals a complex and dynamic iterative process, with components at both the individual and system levels being inevitably inter-related.

**Conclusions:**

The findings emphasise that moral resilience within healthcare organisations is not solely dependent on individual qualities but also influenced by the working groups or teams, leadership and prevailing organisational structures. Supportive interventions should target workgroup dynamics and organisational culture while providing tailored support for individuals.

**STRENGTHS AND LIMITATIONS OF THIS STUDY**

- This grounded theory study combined individual and system-level perspectives, resulting in a conceptual model of the process of moral resilience, including its dimensions, affecting factors and consequences, which can contribute to theoretical development in the field.
- An interprofessional approach, involving a diverse group of informants from general hospital wards, enabled a comprehensive understanding that can inform interventions to support healthcare professionals’ well-being and capacity to deliver high-quality care in both routine and crisis conditions.
- First-person accounts from 46 participants collected through written narratives and interviews provided rich and varied data, strengthening the depth and trustworthiness of the findings.
- A limitation is that data were collected from only two regions in Sweden, which may restrict the generalisability of the findings to other healthcare contexts.

## Introduction

 The COVID-19 pandemic imposed unprecedented moral challenges on healthcare professionals worldwide. Its intensity not only increased the frequency of such challenges but also exposed the limitations of existing support systems. As a result, many professionals experienced moral distress, which significantly impaired their ability to provide compassionate care, a core professional value and negatively affected the overall quality of care.^1^,^3^ The impact on patient care further underscores the urgent need for solutions that support both healthcare professionals and the patients they serve.

Moral distress was introduced as a concept by Jameton in 1984 and is a well-documented phenomenon in healthcare. It is characterised by a state of psychological disequilibrium when individuals perceive a moral obligation to act in a certain way but encounter barriers that prevent them from doing so.^4 5^ Moral challenges may lead to moral distress, which in turn can result in enduring stress reactions and secondary psychological consequences if not adequately addressed.^6 7^ Therefore, it is important to understand how to enable individuals in different positions within the healthcare system to navigate moral challenges in a way that is sustainable both for the individuals and for the complex system as a whole.

Moral resilience, which is essential for mitigating moral distress, enables healthcare professionals to respond effectively to morally challenging situations while upholding ethical standards.^8 9^ For example, moral resilience has been shown to moderate the relationship between moral challenges, such as potentially morally distressing events and moral distress among frontline healthcare professionals.^10^ In this paper, we adhere to Rushton’s definition of moral resilience, which reads *“*the capacity of an individual to sustain or restore [her or his] integrity in response to moral complexity, confusion, distress, or setbacks”*.*^11^ This involves building individual capacity to navigate moral challenges and developing systems that support a culture of ethical practice.^12^ Additionally, a significant aspect of moral distress and moral resilience is that they are phenomena experienced across professions.^13^ To fully comprehend and address moral resilience, a comprehensive approach that integrates diverse healthcare perspectives is essential.

In the aftermath of the pandemic, numerous studies have explored the experiences and consequences of working under extraordinary conditions, including those by Fischer *et al*,^14^ Guttormson *et al*,^15^ Lake *et al*,^16^ Powell and Butler,^17^ Svantesson *et al*,^18^ Sultana *et al*,^19^ Goras *et al*,^20^ Lohela-Karlsson and Condén Mellgren,^21^ and Hedqvist *et al*.^22^ For many in the healthcare sector, the pressured circumstances intensified their moral distress and impacted their well-being, in some cases leading to burnout. These consequences not only affect healthcare professionals but also have profound implications for patient care and outcomes.^3^ Existing literature has primarily focused on the prevalence, context and consequences of moral distress. In contrast, comparatively little attention has been paid to how healthcare professionals developed or employed moral resilience to navigate the moral challenges encountered during the pandemic.

The present study explores moral resilience in general hospital wards, thereby contributing to the broader understanding of resilience during healthcare crises. The aim of the study was to explore the process of moral resilience among frontline healthcare professionals. By delving into their experiences of handling moral challenges during a pandemic crisis, the authors sought to understand the dimensions of moral resilience, affecting factors and consequences. This understanding can inform the design of interventions to support healthcare professionals in sustaining their well-being and delivering high-quality care, both under routine and extreme conditions.

## Methods

### Study design and context

This qualitative study was part of a larger research project entitled ‘Resilient performance in healthcare during the COVID-19 pandemic (ResCOV)’.^2022^,^24^ The project began in 2020, applying an emerging exploratory multilevel design based on grounded theory methodology.

### Setting and participants

Participants in the ResCOV project were recruited from two Swedish regions shortly after the first wave of the pandemic. Healthcare professionals were contacted through their organisations’ email systems where they were informed about the project’s aims, procedures and the voluntary nature of participation. They were also invited to share their experiences during the pandemic and to complete a questionnaire providing demographic data. Informed consent was obtained from all project participants. Open sampling was applied, including all individuals who consented to participate, with the aim of gathering rich data and achieving maximum variation among staff. This strategy resulted in a dataset comprising more than 200 first-person narratives.

The present study focused on healthcare professionals who had been on duty in general hospital wards allocated for patients with COVID-19, either regularly or temporarily, during the first wave of the pandemic. Healthcare professionals working in intensive care units were excluded, as conditions differ between higher-level care and general wards. A purposive sample of participants was drawn from the ResCOV project.^23^ A maximum variation in experiences, occupations and positions was sought, and therefore all available relevant participants were selected to be included.

### Data collection

Data in the ResCOV project were collected from September to November 2020 to retrospectively capture the participants’ experiences from the first pandemic wave, when the situation was still fresh in their minds. At the time of data collection, healthcare professionals were still grappling with the strains of the pandemic. To capture a broad range of experiences without adding to their burden, minimally demanding and flexible data collection methods were employed. Participants could therefore choose to either provide their experiences in writing or in an individual interview.

To facilitate the written narratives and interviews, a study-specific guide was developed,^23^ drawing on a review of publications and the researchers’ experiences (online supplemental file). The guide highlighted specific topics of interest, including working conditions, ethics, patient safety, adaptations, influencing factors, consequences and lessons learnt. The informants were also encouraged to share any additional insights they wished.

The interviews were conducted either face-to-face or digitally by one of the authors (CG or PB-S) and lasted approximately 1 hour on average. All interviews were audio-recorded and transcribed verbatim to ensure accuracy and facilitate detailed analysis.

### Data analysis

Data analysis was guided by the constant comparative method, as described by Strauss and Corbin,^25^ using open, axial and selective coding. To facilitate the organisation and systematic comparison of the data throughout the analysis process, the written narratives and transcribed interviews were imported into NVivo software (V.2020 R1). Theoretical memos were used to track and stimulate coding, reflections and theorisation.

In the open coding phase, data from each informant were read, excerpts related to the aim of the study were identified and codes were inductively derived, starting with the written narratives. Codes from the first analysed narrative were compared, abstracted, merged and organised into preliminary categories, which were successively refined as more data were analysed. Once the preliminary categories were established, the analysis of the interviews focused on identifying additional aspects or clarifications to enhance understanding of the phenomenon, until theoretical saturation was achieved.

In the axial coding phase, the paradigm model—an analytical tool devised to help analysts integrate conditions and context with process^25^—was used. The model assisted in sorting the inductively emerging categories according to process components (contextual, causal and intervening conditions, actions/interactions/reactions and consequences), thereby uncovering the inter-relations between categories. Finally, in the selective coding phase, the categories were further compared and revised until a coherent structure and core category emerged.

The first author (LN) primarily performed the initial open and axial coding, with support from the coauthors (MLK and ECM) at various stages of the process. The last author (PB-S) primarily analysed the interviews, collaborating with the coauthors (CG and MLK), which also facilitated validation of the initial analysis. Additionally, all five authors participated in recurring reflective discussions, selective coding and a final joint revision of the coding until consensus was reached and a theoretical scheme was developed.

In line with grounded theory methodology, theorising was an interpretative process. Such processes can aim for different levels of abstraction, ranging from conceptual ordering to theory creation.^25^ In this study, we aimed at conceptual ordering and created a conceptual model based on the theoretical scheme.

Throughout the study, strategies appropriate for qualitative research were employed to enhance trustworthiness.^26 27^ Consistency in data collection and analysis was ensured through reflective adherence to grounded theory methodology^25^ and the ResCOV project’s approach.^23^ One author had clinical experience as a registered nurse in a general ward during the pandemic (LN), while two others had experience in intensive care nursing (PB-S) and anaesthesia nursing (CG), raising awareness about potential preconceptions influencing the analysis. However, this experience, combined with qualitative research expertise, contributed to theoretical sensibility. Collaboration within the research group, including two coauthors (MLK and EC), without clinical experience from patient care during the pandemic, promoted reflexivity and further enhanced credibility. Dependability was reinforced through theoretical memos, documenting the analytical process and ensuring transparency. Transferability was promoted by contextualising the findings and providing participant quotes (including consecutive participant number), allowing readers to engage with the data.

### Informed consent and data protection

The participants received an information letter emphasising the voluntary nature of participation and their right to withdraw at any time without consequences. Informed consent was obtained from all the participants in the study. Occupational health services were made available to individuals experiencing emotional discomfort. All data were handled in accordance with the General Data Protection Regulation (EU 2016/679) and stored securely.

### Patient and public involvement

None.

## Results

A total of 46 healthcare professionals participated in the study (table 1). The sample represented a wide range of occupations and roles. Both those who worked in their regular positions and those who were in temporary or reallocated roles were represented. In line with the demographics of the study population,^28^ the majority were females. Most participants had more than 5 years’ experience in their profession and over half were under 50 years old.

**Table 1:** Characteristics of participating informants (n=46)

Variable	n (%)
Sex	
Female	39 (85)
Male	7 (15)
Age	
<50	29 (63)
>50	17 (37)
Occupation	
Registered nurse	17 (37)
Assistant nurse	9 (20)
Physician	6 (13)
Manager	5 (11)
Other*	9 (20)
Years in occupation	
≥5	35 (76)
<5	11 (24)
Workplace	
Regular	34 (74)
Temporal	12 (26)
Data source	
Written narrative	37 (80)
Individual interview	9 (20)

* Occupational therapist, physiotherapist, hospital social worker, podiatrist, medical secretary.

**Figure 1:**
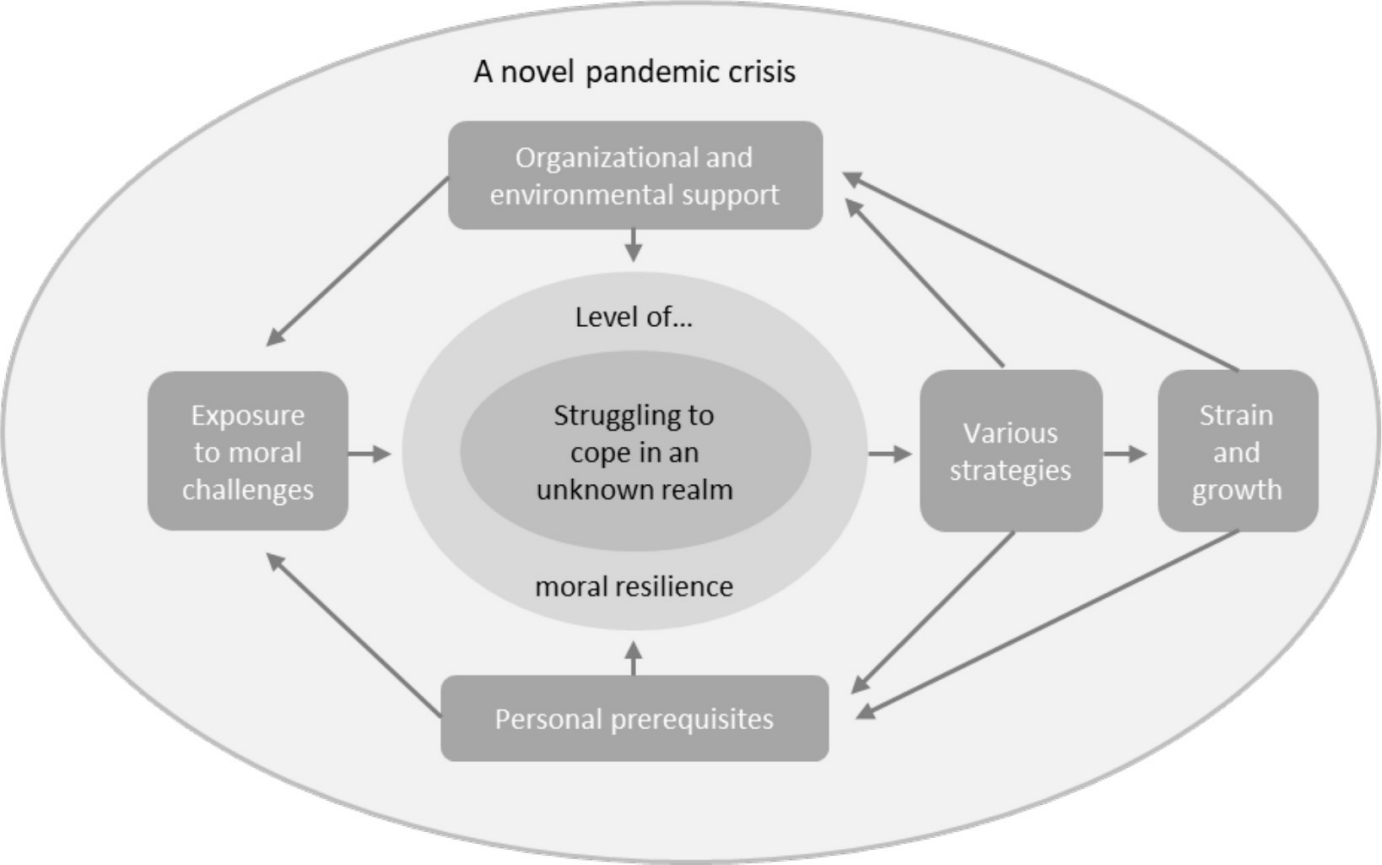
Conceptual model illustrating the process of moral resilience among frontline healthcare professionals, including individual, relational and organisational components and their interrelations.

Based on the informants’ experiences of handling moral challenges during a pandemic crisis, a process of moral resilience among frontline healthcare professionals was illuminated. The findings are presented in a conceptual model (figure 1), accompanied by a table providing an overview of all included categories (table 2). The main text first explains the conceptual model, including the core category, context and the inter-relations between the categories. The main categories are then presented under separate headings, with subcategories in italics. The quotes are provided verbatim, translated from Swedish by the authors, with a parenthetical note indicating the consecutive participant number, data source and the informant’s occupation.

**Table 2:** Core category, main categories and subcategories in the conceptual model of the moral resilience process

Core category—struggling to cope in an unknown realm	
Main categories	Subcategories
Exposure to moral challenges	Conflicting loyalties
	Ethical challenges in clinical practice
Organisational and environmental support	Governance and interpersonal dynamics
	Civil society and private life
Personal prerequisites	Control and confidence
	Meaningfulness and energy
Various strategies	Engagement with management and organisational issues
	Autonomous patient care practice
	Safeguarding oneself to cope
	Suffering in silence
Strain and growth	Impaired occupational well-being
	Development and learning

### The process of moral resilience in the context of a novel pandemic crisis—a conceptual model

The conceptual model developed in this study is grounded in the core category “Struggling to cope in an unknown realm”. This struggle unfolded within the extraordinary circumstances of a novel pandemic crisis and was shaped by varying levels of moral resilience. The process is explained through five interrelated main categories (figure 1), which together with twelve subcategories (table 2), constitute the properties and dimensions of the moral resilience process.

The category ‘Exposure to moral challenges’ conceptualises the immediate causal condition—the starting point or trigger—of the moral resilience process, as illustrated in figure 1. These challenges created an increased need for moral resilience compared with everyday circumstances. As healthcare professionals struggled to cope in an unfamiliar and rapidly evolving context, they experienced and managed moral challenges—including moral adversity—in different ways depending on their level (ie, capacity) of moral resilience.

The individual’s level of moral resilience was shaped by a range of intervening conditions, conceptualised in the categories *‘*Organizational and environmental support’ and ‘Personal prerequisites’. These conditions operated across individual and system levels and fluctuated over time. In their response to their situation, healthcare professionals adopted ‘Various strategies,’ taking actions, interacting and reacting, to handle the moral challenges. These strategies had either a positive or negative impact on both organisational and environmental support and the individuals’ personal prerequisites, that is, the intervening conditions, which further affected their moral resilience in dynamic and iterative ways. Altogether, this dynamic moral resilience process led to both positive and negative consequences for the individuals, as conceptualised in the category ‘Strain and growth’. These consequences included impaired well-being (ie, moral distress or moral injury) in the worst scenario, and development and learning when circumstances were more favourable. In turn, these consequences affected individuals’ prerequisites and their level of moral resilience, thereby contributing to subsequent intervening conditions at the system level.

### A struggle to cope in an unknown realm

The novel contagious disease strained the healthcare system to an extent never before experienced by the informants. The unfolding pandemic forced a massive escalation of healthcare capacity to manage critically ill patients, a task further complicated by pervasive uncertainty and rapid organisational changes. As the system transitioned from routine care, new care facilities opened while others closed, increasing healthcare professionals’ uncertainty due to the disease’s novelty and widespread lack of knowledge. The informants described being plunged into a chaotic situation, with ambition but limited prerequisites to provide ethical care. This tension confronted them with profound moral challenges, captured in the core category ‘struggling to cope in an unknown realm’*.* Several informants likened the situation to working in a war zone, underscoring the extremity and unfamiliarity of the conditions.

It felt like being in a war, you had no idea what was happening, people were dying alone in panic, relatives were afraid to come in and say goodbye, which added even more burden on us to both save lives and try to cope while sitting vigil. (#24, narrative, registered nurse)

### Exposure to moral challenges

The informants encountered a wide range of morally challenging situations, often arising from conflicting loyalties and ethical challenges in clinical practice. These situations had the potential to cause moral distress and tested the healthcare professionals’ ability to maintain their integrity and uphold ethical standards, that is, their moral resilience. How the informants responded to these challenges is further elaborated in the category ‘Various strategies’.

#### Conflicting loyalties

The informants experienced conflicting loyalties between their desire and perceived moral obligation to help patients, their responsibility for routine tasks and their fear of contracting the illness. This tension created a sense of ambivalence, and when reflecting on the moral implications of their actions, some described feelings of remorse. One informant, for example, explained how a moment of compromised adherence to safety procedures during a critical situation led to infection, resulting in sick leave and an inability to contribute to patient care:

I was infected on the ward/…/the patient started vomiting, and the vomit bags were next to me, so I handed one to the patient./…/A personal face shield would have improved the situation/…/my strongest feeling was shame for making such a mistake…. (#20, narrative, registered nurse)

While recognising the importance of conserving resources, informants sometimes experienced guilt when needing to consume supplies, confronting them with what felt like impossible choices. They also described occasions when regulatory principles appeared to overshadow moral considerations within the organisation, leaving them torn between compliance and acting according to what they perceived as ethically right. Informants in both managerial and non-managerial positions reported having the ambition—but limited ability—to lead, support colleagues or influence organisational decisions, which for some resulted in feelings of powerlessness, guilt and moral distress. Additionally, disappointment and concerns about fairness arose when colleagues were perceived as not taking full responsibility during the crisis, which seemed to further intensify the moral challenges.

#### Ethical challenges in clinical practice

Witnessing patients suffer and being unable to cure or fully alleviate their conditions, compounded the ethical challenges the informants faced in clinical practice, at times leading to moral distress. Their strong desire to reduce patient suffering often clashed with the harsh reality, including concerns about patient safety, autonomy and integrity.

The first death occurred in a multi-bed patient room with other patients present. It felt terrible/…/I thought a lot about how I would feel lying there, sick with a completely new disease that no one knows anything about, and they roll out the room neighbor who has died from that exact disease./…/Therefore, we tried placing palliative care patients in a monitoring room, for some peace and quiet. This also didn’t feel ethically right to us, as the room was empty and bare. It was so obvious what was going to happen to the patient. It felt terrible. We made many attempts to make it as dignified as possible for these patients, but nothing felt okay. (#29, narrative, medical secretary)

Strained circumstances forced difficult prioritisation, and informants described feeling lost and helpless when even trusted sources of medical guidance lacked answers. They witnessed compromised patient safety and sometimes questioned whether more assertive advocacy could have mitigated avoidable patient harm or even saved lives, generating further ethical concerns. Feeling responsible for their patients, several expressed that healthcare should have been able to provide better care, which deepened their moral challenges.

This naturally caused anxiety and stomach aches among the staff and was perceived as unsafe for the patients. Our patients were significantly marginalized during this period, and I didn’t feel like we were being heard, even though most of them were truly suffering. (#39, narrative, registered nurse)

Ambiguities regarding levels of care were perceived to delay intensive care responses, and discharges were experienced as rushed. Deficiencies in palliative care expertise and overall patient management were exacerbated by medication shortages, inadequate staffing and limited access to personal protective equipment (PPE), all of which necessitated adaptations that felt ethically unfavourable. Visitation restrictions heightened the distress of isolated patients, many of whom died alone. Time constraints, infection control measures and physical barriers further reduced opportunities for patient contact, challenging the informants’ ability to offer adequate support.

Taken together, these conditions contributed to substantial ethical strain in clinical practice, intensifying the moral challenges experienced by the healthcare professionals.

### Organisational and environmental support

The healthcare professionals’ experiences and their ability to manage moral challenges, that is, their moral resilience, were strongly influenced by the organisational and environmental support available to them. This support could either bolster or undermine their capacity for moral resilience and thereby shape the responses and strategies they adopted. The support was related to organisational factors, such as governance and interpersonal dynamics, as well as influences from the environment outside work, including civil society and the private life of the informants.

#### Governance and interpersonal dynamics

Rapid changes, exclusion from decision-making, feeling undervalued and managers overwhelmed by administrative demands led to feelings of abandonment and of being ‘like a pawn in a game’ (#5, narrative, registered nurse). Such experiences highlighted insufficient support at various leadership levels and undermined moral resilience.

Abrupt reassignments, whether voluntary or imposed, to unfamiliar workplaces created insecurity and a sense of displacement, described by one informant as feeling ‘homeless and lost’ (#10, narrative, registered nurse). Inappropriate care facilities, heavy workloads, limited introductions to new units, insufficient team competence and shortages of both material and human resources heightened uncertainty and further weakened the capacity for moral resilience.

At the same time, a strong sense of teamwork and solidarity emerged among healthcare professionals across disciplines, driven by the clear crisis and the need for collaboration. This strengthened sense of fellowship was perceived as inspiring and appeared to reinforce moral resilience.

The collaboration between physicians from different clinics and between professionals was incredibly inspiring. Everyone wanted to help each other, and there was no ‘not my patient’ mentality that I encountered. Even in contact with other hospitals, everyone wanted the best for the patients and understood that we were not sufficient physically or in terms of competence. (#23, narrative, physician)

The mix of experiences and competencies fostered effective problem-solving and contributed to staffing stability, which was reassuring. Adequate staffing, together with supportive colleagues and managers, provided a sense of security and was viewed as essential for problem solving and flexibility in the chaotic situation, and the maintenance of moral resilience.

I think a success-factor has been that decisions were made quickly, and measures were swiftly taken to enable staff layoffs. Having managers in place who were tasked with solving this problem and fostering a spirit of cooperation, where everyone pitched in with ‘Now we solve this’. I believe that that has been the key. (#3, interview, registered nurse)

Keeping up with rapidly changing information was challenging and contributed to ethical strain. Clear and timely communication was considered critical for maintaining a sense of security. This highlighted the need for clear protocols and supportive teamwork to provide a safe space and foster moral resilience.

Despite daily staff meetings, physicians were often better informed than nursing staff, contributing to feelings of disparity and undermining moral resilience. Over time, however, the establishment of clearer rules, procedures and improved knowledge appeared to support moral resilience and enhance the capacity to navigate morally challenging situations.

#### Civil society and private life

Environmental influences from civil society and the informants’ private lives had a substantial impact on their experiences and capacity for moral resilience, shaping their ability to navigate the unprecedented moral challenges.

Extensive media coverage and the continuous influx of pandemic-related updates meant that healthcare professionals were exposed to the crisis even during their free time. This reduced opportunities for recovery and appeared to weaken their moral resilience. Conversely, positive media attention and appreciation from civil society played a notable role in bolstering moral resilience and strengthening their sense of purpose.

Informants also described how their private lives either intensified or alleviated strain. Responsibilities at home and concerns about the health and well-being of family members contributed to a sense of burden and sometimes created conflicting loyalties. In contrast, having a supportive network of family and friends was perceived as relieving and appeared to facilitate moral resilience, as summarised by one informant:

…you need a good social network in your private life to get some distance. Therefore, you can air your thoughts or replenish them with other things. To be able to be an ordinary human being sometimes because it was very intense. (#9, interview, manager/registered nurse)

### Personal prerequisites

In addition to the system-level intervening conditions, the healthcare professionals’ ability to manage moral challenges was strongly shaped by their personal prerequisites. These included their sense of control and confidence, as well as their experiences of meaningfulness and energy. Together, these factors either facilitated or hindered their capacity for moral resilience.

#### Control and confidence

The informants’ sense of control and confidence varied considerably. Some felt able to influence situations, contribute meaningfully to teamwork and rely on their professional self-confidence—all of which appeared to promote their moral resilience.

…I wasn’t thrown into something I didn’t agree to, in any way, myself. In addition, I also felt that… yes, but I’ve been a manager for so many years, I know what I’m doing… (#1, interview, ward manager/registered nurse)

Those who initially felt empowered and confident seemed able to maintain this sense of assurance throughout the pandemic. However, others experienced powerlessness and uncertainty, combined with a perceived inability to influence their work situation or work-life balance, which seemed to weaken their moral resilience.

…There wasn’t enough time to care for patients and their families adequately, and dementia patients were left alone in rooms with severe anxiety. I feel I was insufficient in many situations, and the uncertainty of our work stressed me out… (#18, narrative, nursing assistant)

Fear of contracting the virus, together with knowledge gaps in the early stages of the pandemic, intensified negative emotions and contributed to feelings of insecurity and frustration. Additionally, some informants were open and receptive to seeking and accepting support, which appeared to strengthen their moral resilience, whereas others were less inclined to seek support and thus appeared more vulnerable to moral distress. As guidelines and knowledge improved over time, some began to regain confidence, while others continued to struggle with feelings of vulnerability, indicating a diminished capacity for moral resilience.

#### Meaningfulness and energy

The informants’ sense of meaningfulness at work, along with their levels of energy, varied considerably. Meaningfulness was closely linked to loyalty, dedication, curiosity and interest, as well as to the ability to find purpose in one’s professional role to fulfil a purpose.

I would say that those first weeks were… that’s when I truly worked a lot of overtime, plus the time I worked was constantly stressful because you wanted to do it as well as possible and you felt like ‘What I’m doing actually matters’. (#13, interview, physician)

A strong sense of meaningfulness appeared to facilitate moral resilience. However, while a heightened sense of responsibility provided purpose for some informants, it also increased stress for others, for example, among those who worried about delayed routine tasks that had been put aside, which gradually drained their energy. Some informants also expressed frustration over what they perceived as unnecessary reassignments and departmental changes. For others, forming strong emotional bonds with patients depleted their energy and contributed to fatigue and exhaustion. Altogether, these factors appeared to undermine moral resilience.

Conversely, positive personality traits and the ability to use humour were described as helping make the situation more bearable and were thus interpreted as contributing to enhanced moral resilience.

### Various strategies

In their struggle to cope in an unknown realm, the informants navigated moral challenges by employing various strategies. These strategies were either deliberate or unconscious, and they evolved over time depending on the situation. Influenced by the previously described intervening conditions, the strategies ranged from passive to proactive responses and operated across different system levels. They included engagement with management and organisational issues, autonomous patient care practices, safeguarding oneself to cope and suffering in silence. As elaborated below, the effectiveness of these strategies varied. Some informants appeared able to maintain their integrity and uphold ethical standards more successfully than others. This variation was interpreted as reflecting different levels—or capacities—of moral resilience among the informants.

#### Engagement with management and organisational issues

Engagement with management and organisational issues encompassed initiatives such as volunteering across wards, actively contributing to organisational structures and sharing essential information. These efforts illustrated healthcare professionals’ commitment to meeting organisational needs and enhancing overall effectiveness, while simultaneously fostering personal satisfaction and a sense of meaningfulness. Such engagement was also highly appreciated by colleagues.

…a colleague did an excellent job creating structure. In addition, said, ‘okay, here’s where I’m needed, I’ll take responsibility’. And he was the one who also ensured from the medical side that there was stable staffing. (#7, interview, physician)

Healthcare professionals actively supported colleagues by drawing on their diverse skills, training and experiences, which fostered a collaborative and supportive environment. Engaging in dialogue, advocating for tailored palliative care and contributing to the dissemination of information were commonly described as important ways of managing daily challenges and supporting the team. Initiatives that promoted interprofessional conversations and open communication allowed team members to share insights and strategies, thereby strengthening organisational support and, by extension, their moral resilience.

Strategies for expressing dissent, whether concerning organisational issues or personal challenges, included raising concerns with managers, negotiating work conditions and seeking support from trade unions. However, these efforts were largely perceived as ineffective. When attempts to influence decisions or obtain support were met with limited responsiveness, several informants described feelings of abandonment or betrayal, which appeared to undermine their moral resilience.

I tried to seek help from my regular unit manager and operational manager. My unit manager repeatedly said she couldn’t do anything. Or that she’s doing the best she can… I felt so betrayed by the leadership… (#46, narrative, nursing assistant)

#### Autonomous patient care practices

The informants employed a range of innovative and autonomous patient care practices to navigate moral challenges, overcome obstacles and uphold ethical standards. To reduce patient suffering, they developed alternative communication methods that enabled patients to stay in contact with their families. In some instances, they assumed responsibilities beyond their usual roles—such as assisting patients with legal matters, including preparing wills. These actions appeared to help restore their sense of integrity and were interpreted as strengthening their moral resilience.

For the patient, it was crucial to write a will/…/I didn’t know how to proceed, but then I decided to call the hospital lawyer, who supported me by explaining there were exceptions in the law for the current situation. (#4, narrative, social worker)

The informants also frequently spoke up and advocated for patients’ best interests, often emphasising the need for individualised palliative care. Many chose to forgo scheduled breaks to provide more attentive care, and some willingly risked exposure to contagion during emergencies to protect others. At times, they deviated from established rules when these were perceived as conflicting with patients’ dignity or well-being—for example, by facilitating family visits despite restrictions. Such decisions seemed to reflect a commitment to their moral or ethical compass and were interpreted as expressions of moral resilience.

…we did make exceptions to the visitation ban/…/No one should have to die alone. (#10, narrative, registered nurse)

#### Safeguarding oneself to cope

The informants employed a range of strategies to safeguard themselves and cope with the moral challenges and demanding conditions they faced. These strategies were interpreted as reflecting varying levels of self-awareness and self-regulation. Many engaged in continuous learning to stay informed and maintain a sense of control in the rapidly evolving situation. To prevent burnout, some deliberately protected their recovery time by declining extra shifts and engaging in restorative activities such as spending time with social networks, exercising or simply resting. Others actively reduced their responsibilities to manage stress and workload—for example, by taking on tasks that did not require extensive decision-making.

I eventually requested that I just be placed as a nurse on the ward to avoid having to do rounds, have conversations/dialogues with relatives, etc., because it was more than I could handle. (#25, narrative, registered nurse)

To preserve their well-being, some informants also described compartmentalising their emotions to regulate empathy and mitigate excessive emotional strain. This often involved limiting interactions with patients or relatives to maintain professional boundaries and emotional stability. Such emotional distancing appeared to help them uphold their integrity and continue providing effective care. Safeguarding strategies also included delegating tasks, concentrating on core responsibilities and intentionally minimising engagement with issues beyond their control. By focusing on defined responsibilities, they could remain effective while reducing moral strain.

Humour and celebrating small achievements emerged as additional coping mechanisms, helping to foster a sense of cohesion and psychological relief:

I would say that we had a nice attitude. In the middle of the situation, we could just like “Hello, what kind of chaos is this?” And then we laughed for a while, and then just “no, but now we’re moving on”. (#1, interview, ward manager/registered nurse)

Maintaining a positive outlook was supported by efforts to reframe situations constructively and remain focused on their core mission. Conversely, some informants attempted to shield themselves from moral obligation by attributing responsibility for the challenges to the organisation or managers. However, this strategy seemed to contribute more to increased frustration than the intended relief.

#### Suffering in silence

In contrast to the more proactive strategies described above, some informants appeared to adopt a passive approach, enduring moral challenges and moral distress without actively addressing the situations. By ‘suffering in silence’, they struggled to articulate concerns or seek support, which gradually eroded their sense of integrity and well-being and hindered their ability to uphold ethical standards. Several continued working in temporary settings despite persistent uncertainty about their competence or the overall quality of care being provided.

… the communication and expectations of what should be done were very different. This made me feel very alone and even more inadequate. This is not something that the clinic management/managers have failed, it is entirely my own approach and feelings. Since there was very little time for reflection/debriefing and I felt that I was just working on and doing the best I could, my own feelings and all the ethical dilemmas I had faced caught up with me later. (#26, narrative, registered nurse)

Due to time constraints, feelings of hopelessness, insecurity or a lack of emotional energy, these informants refrained from seeking support. By internalising their emotions and attributing challenges to personal shortcomings, they intensified their vulnerability to moral distress. In some cases, this pattern appeared to progress toward a more severe state, resembling moral injury, where the accumulation of unaddressed moral adversity overwhelmed their capacity for moral resilience.

#### Strain and growth

Overall, the struggle to cope in an unfamiliar realm and the process of navigating moral challenges reflected the ongoing development of the informants’ capacity for moral resilience. Individuals actively worked to preserve their integrity and uphold ethical standards, yet these efforts resulted in both positive and negative consequences, including experiences of ‘strain and growth’. The strain and growth were manifested in impaired occupational well-being as well as personal development and learning. These consequences often coexisted and were not strict opposites. However, some individuals leaned more toward one end of the spectrum depending on their exposure to moral adversity and their capacity for moral resilience. These perceived consequences, in turn, shaped the development of their personal prerequisites and influenced the broader intervening conditions at the system level.

##### Impaired occupational well-being

The struggle to cope in an unknown realm took a clear toll on informants’ well-being, manifesting in both physical and psychological symptoms. At times, the intensity of the moral challenges appeared to exceed their capacity to respond sustainably, resulting in sleep disturbances, emotional exhaustion, burnout, depressive symptoms and negative self-evaluations of professional competence. For some, the experience led to a firm resolve to avoid similar situations in the future, while others described symptoms indicative of post-traumatic stress. These impairments occasionally led to extended sick leave, profound disappointment and intentions to leave their roles.

What I personally take away from this is that I’ve become more determined about what I don’t want to experience again. I feel too old for this, to go through it one more time. Not to make an effort, but to be just a pawn in a game. (#27, narrative, nursing assistant)

For several informants, strain persisted beyond the first wave of the pandemic, and uncertainties about the future appeared to diminish their sense of moral resilience.

##### Development and learning

Alongside the hardships they faced, informants also described significant personal development and learning, which contributed to experiences of growth. Many expressed both pride and sorrow–sorrow over the suffering and losses they had witnessed, and pride in what they and their colleagues had accomplished under extreme circumstances. These reflections appeared to bolster their moral resilience by reinforcing a sense of purpose and professional identity.

When I look back on the work, I do so with both pride and sorrow. Sorrow over knowing that patients died alone in wards without the possibility of having staff or relatives with them. However, also, with pride over the work everyone did/…/. (#42, narrative, occupational therapist)

New experiences, diverse team constellations and knowledge sharing enhanced competence and confidence, strengthening informants’ readiness to face future challenges. The extensive need for rapid adaptation and decision-making fostered creativity and learning, while collaboration in new teams broadened their skills and perspectives. Many described emerging from the first pandemic wave stronger, more self-assured and more attuned to their own ethical commitments. They also found reassurance in the organisational resilience demonstrated by the healthcare system during the crisis, which was perceived as a positive outcome of the experience.

It was amazing to see how decision-making processes and all adjustments, which normally can be as long as anything, were shortened to almost nothing. It works! (#40, narrative, physiotherapist)

Functioning as an infectious disease physician in a leadership role during the COVID-19 outbreak has undoubtedly been a significant challenge in many ways (especially in terms of workload, particularly in the early days). However, it has also been very satisfying and enlightening to be part of this experience. (#30, narrative, physician)

## Discussion

The main result of this study is a conceptual model that describes and explains the process of moral resilience among frontline healthcare professionals in general hospital wards during a pandemic crisis. The model illustrates a complex and dynamic iterative process, in which individual and system-level components are closely interdependent. This interdependence can be understood as reflecting a synergistic relationship between individual moral capacities and the ethical practice environment (cf. 29).

The findings contribute to existing knowledge by offering empirically grounded insights into multiple dimensions of moral resilience, its consequences and influencing factors. The model aligns with the key elements of moral resilience highlighted by Rushton *et al*^29^—personal and relational integrity as the ethical anchor, self-regulation and self-awareness supporting the individuals response to moral stressors, and moral efficacy referring to the confidence in one’s capacity to act in accordance with moral commitments despite adversity. At the same time, the model incorporates context-specific categories grounded in frontline professionals’ experiences during the COVID-19 pandemic, thereby extending and nuancing existing theoretical work. Particularly, the dynamic, process-oriented character of the phenomenon and the interplay between individual capacities and relational or organisational conditions are displayed.

Although this study focuses on general hospital wards, many of the challenges described by informants resemble issues widely reported across healthcare settings during the pandemic.^30 31^ This congruence suggests that the moral adversities and coping strategies identified here reflect broader patterns among healthcare professionals during the pandemic crisis. Moral resilience is consistently associated with lower distress and better mental health across professions, whereas exposures such as redeployment and end-of-life constraints are linked to adverse outcomes.^1032^,^34^ Additionally, post-peak analyses in critical care settings further underscore that addressing institutional factors—such as perceived betrayal or unsafe staffing—is indispensable; moral resilience may buffer distress, but it cannot substitute for necessary systemic remedies.^10^

The findings underscore that moral adversity is not merely an individual phenomenon, but is deeply shaped by systemic, organisational and policy-level conditions—highlighting the need to foster moral resilience through structural as well as individual and relational interventions. While moral challenges and moral distress are experienced at the individual level, many of the determinants identified in our study are structural. Informants described rapid redeployments, information asymmetries and shortages of critical resources (eg, PPE, staffing, medications), combined with limited opportunity to influence swift decision-making processes. Such conditions are well-recognised external drivers of moral distress: institutional failures related to inclusion, transparency and support can erode integrity and heighten vulnerability to moral adversity. In particular, experiences of institutional betrayal have been associated with elevated risk of moral distress, especially when system-level shortcomings remain unaddressed and organisational resilience is insufficient.^32^

### Dimensions and consequences

As indicated by the substantial body of research on COVID-19,^31^ the present findings show that the multitude of morally challenging situations required healthcare professionals to develop or draw on their moral resilience to navigate the crisis in a sustainable manner. Moral resilience manifested through a variety of strategies, which had either beneficial or detrimental consequences for their ability to maintain integrity, uphold ethical standards in care and protect their own well-being.

The strategies ranged from passive to proactive responses and operated across different system levels. For example, the strategy of suffering in silence eroded individuals’ integrity over time. Some individualistic strategies—such as raising concerns for personal benefit—were more proactive but not consistently effective. Other proactive responses included safeguarding one’s well-being, ensuring opportunities for recovery and mobilising strategies to navigate moral challenges for the benefit of patients, teams or the broader organisations.

These self-protective approaches align with Rushton’s notion of self-stewardship, emphasising intentional practices to sustain well-being and moral integrity in ethically demanding clinical contexts.^29^ Strategies characterised by team collaboration and readiness to intervene when needed were often perceived as more successful. Such collective approaches reflect what Rushton terms relational integrity, that is, acting in ways that uphold both one’s own and others’ moral commitments within the clinical environment.^29^

Thus, the findings align with Osifeso *et al*,^35^ who emphasise that strengthening moral resilience involves both individual-level components—such as self-care, self-regulation and moral courage—and group-level components, such as peer support. Communication emerged as a key strategy across both levels. These relational strategies resonate with Rushton’s work, which highlights the value of psychologically safe spaces for dialogue, structured ethical support and peer reflection as collective practices that help preserve integrity and bolster moral resilience.^36^

Insufficient moral resilience capacity led to moral distress and, in some cases, to a more corrosive form of distress that threatens integrity—moral injury.^37^ Indicators of impaired occupational well-being included sleep disturbances, emotional exhaustion, depressive symptoms, burnout and a diminished sense of professional efficacy. In some cases, as also found in other studies,^3038^,^40^ these consequences resulted in long-term sick leave or intentions to leave the profession, affecting both individuals and the healthcare system.

Conversely, when informants described having access to resources and support that enabled them to navigate moral challenges, this was sometimes associated with personal development and growth—even in the midst of undeniable moral strain. This finding aligns with previous research showing that certain individuals possess protective factors that buffer against moral distress.^41 42^ Recent studies further demonstrate that moral resilience functions as a protective resource: higher levels of moral resilience are associated with reduced burnout and turnover intention,^43^ lower levels of moral injury^33 34^ and attenuated effects of distressing events on moral distress.^10^ It is also noteworthy that individuals in the same environment may exhibit varying levels of moral resilience.^32^ Moreover, the capacity for moral resilience fluctuated over time, depending on situational demands and the conditions affecting it. Together, these findings reinforce the view that moral resilience is context-specific, and that an individual may demonstrate considerable moral resilience in one situation but not in another.^44^

### Factors promoting moral resilience

As also illustrated in the present conceptual model, factors influencing an individual’s capacity for moral resilience encompass both personal prerequisites and organisational and environmental support.^45^ Although this qualitative study did not include any formal measurement of levels, these factors appeared to exist along a continuum, ranging from less to more favourable conditions for moral resilience. Drawing on the informants’ experiences, it was evident that the greater the presence of factors situated toward the advantageous end of this continuum at the time of a moral challenge, the stronger the individual’s capacity for moral resilience. This aligns with previous research emphasising the complex and multifactorial origins of moral distress. Rushton,^45^ for example, argues that moral distress may stem from internal factors—such as feelings of powerlessness—as well as external factors, including resource shortages, staffing constraints and inadequate organisational support. Altogether, these findings underscore the importance of both personal and relational integrity.^12 46^

At the individual level, several factors were identified as facilitators of moral resilience: a sense of meaningfulness, the ability to see purpose in one’s role, contributions to teamwork, self-confidence and feelings of empowerment. Loyalty, dedication, curiosity, interest, responsibility, humour and positive personal traits were all linked to a sense of meaningfulness. These categories map well onto Rushton *et al*’s^29^ key attributes of moral resilience—integrity, moral efficacy and self-regulation—suggesting that the individual facilitators identified in this study reflect core components of the construct. For instance, meaningfulness and purpose relate to moral integrity; teamwork contributions and empowerment reflect moral efficacy, while humour and positive traits may support emotional self-regulation. Based on the multiple interacting factors influencing moral resilience during the explored crisis, the findings also resonate with the non-dualistic approach proposed by Kuldas and Foody,^47^ who argue that moral resilience encompasses both inherent personality traits developed over time and dynamic states that individuals can cultivate in response to specific situations.

According to Rushton,^36^ moral resilience involves developing skills and methods that empower professionals to deepen their connection to core values, recognise their moral obligations and effectively navigate ethically complex scenarios. This notion resonates with experiences revealed in our study. Rushton^45^ further emphasises the need for individuals to alter their relationship to the suffering generated by moral distress. This dynamic nature of moral resilience suggests that individuals who do not initially possess inherent traits facilitating moral resilience may still cultivate protective qualities through targeted interventions and supportive environments. While Rushton’s early work focuses primarily on nurses, later studies expand the scope to interprofessional contexts.^10 35 37 44^ These contributions, together with insights from the present study, suggest that these notions are relevant from an interdisciplinary perspective and across various healthcare professions.

At the group level, teamwork and collaboration, collegial support, solidarity and diversity in experiences were all linked to moral resilience. These elements resonate with Rushton’s concept of relational integrity, a team-based dimension of moral resilience characterised by the collective alignment of values, intentions and behaviours within interprofessional teams and organisations.^29^ Support from family, friends and civil society—such as positive media attention and public appreciation—also contributed to strengthened moral resilience. Previous research similarly highlights the importance of supportive workplace environments,^48^ strong social support networks^41^ and adaptive coping strategies,^49^ as buffers against stress and moral distress. Moreover, Maunder *et al*^50^ argue that while adaptive skills may benefit individuals under prolonged occupational stress, recovery depends equally on organisational or environmental factors. These works are further supported by results in the present study—both in terms of the importance of targeted interventions and supportive environment to nurture moral resilience, as well as its relevance for a variety of healthcare professions.

At the organisational level, supportive managers, staffing stability, clear communication and established procedures—as well as opportunities to influence decisions—appeared to foster moral resilience. This aligns with prior research showing that feeling supported or betrayed by leadership is a crucial determinant of moral resilience.^15^,^1832^ Managers and physicians in the present study did not appear to encounter the same levels of uncertainty and lack of control as nursing staff. One plausible explanation is that shorter communication pathways and closer involvement in decision-making provided clearer information and a stronger sense of agency. This interpretation is consistent with recent findings showing that organisational structures ensuring, for example, transparency and meaningful involvement in decision-making are central to strengthening moral resilience and reducing moral injury among healthcare leaders.^34^

### Implications for healthcare practice

Based on the current research, the moral resilient process illuminated in the present study can be promoted by addressing the range of intervening conditions. Establishing a supportive culture in daily practice can enhance preparedness for managing complex future situations and crises.^51^ Intervention programmes that integrate individual-level, team-level and organisational-level components may effectively support healthcare professionals and strengthen moral resilience across the system.

At the individual level, relevant components include stress-management and self-regulation practices—such as mindfulness or meditation—to support emotional regulation, as well as ethics education aimed at strengthening ethical discernment, moral awareness and the ability to translate values into action when facing morally complex situations.^29^ Additionally, so-called self-stewardship is relevant, meaning a commitment to know oneself, to choose actions that are wholesome and healthy and to commit to taking action to support one’s own well-being while compassionately honouring one’s limitations through.^29^

At the team or group level, peer support can facilitate the processing of potentially traumatic events, mitigate stress and improve overall occupational well-being.^51^ Collective reflection on moral challenges may promote adaptive responses and buffer against moral distress.^2^ Additionally, fostering engagement among peers, teams and managers—while ensuring a psychologically safe work environment—is crucial.^52^ Investing in such strategies has the potential to strengthen both individual resilience and organisational commitment, even under conditions of limited resources.^48^

At the organisational level, measures may include strategic scenario planning and training that prepare staff to handle unexpected events and crises through simulated scenarios and contingency plans.^49^ It is also important that such planning incorporates structures that enable meaningful employee participation in decision-making processes.^53^ Clear policies for resource allocation, transparent processes for reporting safety or ethically challenging events and having dedicated resources to support staff to address ethical challenges is pivotal. Furthermore, supporting managers in developing effective leadership is essential. This includes fostering open and structured communication, valuing each team member’s contribution, involving staff in decision-making and promoting a healthy work-life balance.

Overall, our findings reinforce a central conclusion regarding the fostering of moral resilience: the burden of transforming moral distress cannot rest on individuals alone. Cultivating moral resilience requires coherent action across all levels—individual, team, organisational and system—to create the conditions under which healthcare professionals can preserve or restore integrity and deliver ethical, high-quality care, both in routine practice and in times of crisis.^36^

### Methodological strengths and limitations

The qualitative explorative design, based on grounded theory methodology,^25^ provided valuable insight into the dynamic process of moral resilience during a pandemic crisis, resulting in a conceptual model of the phenomenon. Grounded theory research can be directed toward different levels of abstraction, ranging from conceptual ordering to theory creation.^25^ This study aimed at conceptual ordering. Nevertheless, the developed conceptual model demonstrated explanatory power within the substantive area explored, suggesting that it may be regarded as a substantive theory.

A key strength of this study is its combined individual-level and system-level perspective, as well as its interprofessional approach. By including a diverse group of frontline healthcare professionals from general hospital wards in two different regions, the study captured a wide range of perspectives and strategies used to navigate moral challenges. This diversity facilitated a comprehensive exploration of different dimensions of moral resilience, its consequences and the factors influencing it.

In grounded theory research, data collection and analysis are typically conducted simultaneously. In this study, data were drawn from a purposive sample of participants in the ResCOV project,^23^ which introduced certain limitations. The recruitment method—relying on organisational email systems—may have led to sampling bias by potentially excluding individuals who did not regularly check their emails. However, to accommodate different preferences, participants were given the option to share their experiences either in writing or through individual interviews. In grounded theory, both those methods are considered to be analytically valuable forms of data, consistent with the methodology’s emphasis on flexibility and the inclusion of any material that supports the development of emerging categories.^25^ This approach resulted in a substantial dataset, encompassing insights from more than 200 informants.

Data were collected retrospectively, which introduces a risk of recall bias. Additionally, during the constant comparative analysis, it was not possible to follow-up with informants for clarification or adjust subsequent interviews based on emerging insights. However, as participants were approached soon after the studied event, their accounts remained vivid and the often-detailed descriptions suggest that the experience was still fresh in their minds. This contributed to the richness and depth of the data.

Using Strauss and Corbin’s paradigm model^25^ as an analytical tool provided a structured approach that facilitated the exploration of the process in the inductive analysis. However, researchers’ preconceived notions and theoretical preferences inevitably influence interpretation, introducing a risk of bias. To mitigate this, reflective discussions were conducted within the author group, which included researchers from diverse disciplines and with varied research backgrounds.

Finally, a key limitation of the study is that only qualitative data were included. A mixed-methods design could have enabled triangulation, for example, by incorporating quantitative data from validated instruments.

## Conclusions

This study presents a conceptual model describing the process of moral resilience among frontline healthcare professionals working in general hospital wards during a pandemic crisis. By offering a multilevel perspective, the findings deepen the understanding of the dimensions, consequences and influencing factors of moral resilience. The results highlight that moral resilience is shaped not only by individual capacities but also by relational and organisational conditions that together influence how individuals respond to moral challenges.^1^

Adopting a systemic perspective is essential for integrating these components and identifying organisational factors that can strengthen moral resilience across the healthcare system. Such knowledge is crucial for designing interventions that support moral resilience not only during crises but also under routine conditions, thereby promoting long-term well-being and the capacity to deliver high-quality care. Effective interventions should address workgroup dynamics, organisational culture and tailored individual support.

Furthermore, understanding the role of leadership in fostering resilience and supporting healthcare professionals during crises is vital. Future research should explore leadership strategies in greater depth to equip decision-makers with practical tools to address moral challenges in healthcare.

## Supplementary material

10.1136/bmjopen-2025-104065 online supplemental file 1

## Data Availability

Data are available upon reasonable request.
