# Supplementary material for: Struggling to cope in an unknown realm: a qualitative study of moral resilience in frontline healthcare professionals in Swedish hospital wards during a pandemic crisis
Source: BMJ Open. 2026 Mar 23;16(3):e104065. doi: 10.1136/bmjopen-2025-104065 (PMC13034357; doi:10.1136/bmjopen-2025-104065)
Supplement: online supplemental file 1 [file bmjopen-16-3-s001.pdf]

This is a translation from Swedish to English, completed by AI/ChatGPT on 1/31/2025.

Interview Pre-Survey

The research project aims to learn from the transition that healthcare has made due to Covid-19. You may choose what you wish to share. All information will be compiled at a group level so that no individual can be identified, and no confidential information will be disclosed. More information about the study and contact details for the responsible researchers for any questions can be found in the email you received.

Please save the survey on a computer where unauthorized persons cannot access it and fill in the background questions below (it's fine if it takes up several pages).

If you have documents, such as procedures, guidelines, and decision logs that can help us understand your story, feel free to attach them with your response.

When you are finished, please send the survey, the consent form, and any other documents to: **[e-mail].**
If we have scheduled an in-person meeting for the interview, you may bring the documents to the meeting.

Thank you in advance!

Background Questions

1. Gender: …
2. Age: …
3. Profession: …
4. Years in this profession: …
5. Regular workplace, role, and job responsibilities: …
6. Workplace, role, and job responsibilities during the pandemic: …
7. May we contact you for any further questions? …

Contact details: …

This is a translation from Swedish to English, completed by AI/ChatGPT on 1/31/2025.

My Experience from Covid-19: Support Guide for Your Story

The research project aims to learn from the transition that healthcare has made due to Covid-19, focusing on adaptability, patient safety, work environment, and ethics. By submitting your story, you consent to participate in the study. More information about the study and contact details for the responsible researchers for any questions can be found in the email you received.

You may choose what you wish to share, but you can use the questions on the next page as support.

Please save the document on a computer where unauthorized persons cannot access it. Fill in the background questions and your story. If you have documents, such as procedures, guidelines, and decision logs that can help us understand your story, feel free to attach them with your response.

When you are finished, please send your story and any other documents to:
[e-mail].

All information will be compiled at a group level so that no individual can be identified, and no confidential information will be disclosed.

Background Questions

1. Gender: …
2. Age: …
3. Profession: …
4. Years in this profession: …
5. Regular workplace, role, and job responsibilities: …
6. Workplace, role, and job responsibilities during the pandemic: …
7. May we contact you for any further questions? …

Contact details: …

What Should I Share?

The focus of this study is on adaptations, influencing factors and consequences, patient safety, work environment, and ethics. We are interested in conditions/opportunities and vulnerabilities within everything from organization and working conditions, equipment, materials, premises, and resources to management/governance, employee engagement, knowledge/competence, support, working methods, collaboration, information and communication, perceptions of patient safety, patient encounters, and contact with relatives, as well as your personal feelings and reflections. You may choose what and how much or little you wish to share.

1. Please share in your own words your experiences and thoughts from the transition in the spring and the current situation—both positive and negative. Concrete examples

are welcome.

2. After writing down your story, you may check against the following questions to see if there is anything you wish to add:
   - How have the demands on healthcare been managed? What did you do? How did it go? What was difficult? How did you succeed? Why? Who contributed? Have decisions been made and followed? Adaptations in care and decisions? How has the situation been controlled? Learning over time? Anticipation? What weaknesses and strengths have influenced the changes? Consequences/results (desired/unwanted)?
   - How have you personally managed the transition, been affected, and acted? Work situation? Ethical dilemmas? Existential thoughts? What have you done to cope with the situation? What has helped? What has made it more difficult? Consequences for you?
   - Ideas for the future? Success factors to carry with us? Anything else you wish to share?

Please note that the above questions are only a guide; you share what you want and consider relevant based on your role in the transition of healthcare during the Covid-19 pandemic. If you have documents, such as procedures, guidelines, and decision logs that can help us understand your story, feel free to attach them with your response.

My Story:

(You can write an unlimited number of pages)

This is a translation from Swedish to English, completed by AI/ChatGPT on 1/31/2025.

My Experience from Covid-19: Interview Guide

Thank you for participating. We will audio record, transcribe, and anonymize the recording before analysis. All information will be compiled at a group level so that no individual can be identified, and no confidential information will be disclosed. You may choose what and how much or little you wish to share. Participation is voluntary and can be terminated without reason.
(Contact details for occupational health services will be provided after the interview.)

Additional documents:

- Consent Form
- Background Questions (Survey)

The research project aims to learn from the transition that healthcare has made due to Covid-19, focusing on adaptations, influencing factors and consequences, patient safety, work environment, and ethics.

We wish to hear your experiences and thoughts from the transition in the spring and the current situation—both positive and negative—with concrete examples.

Main Questions

- Can you start by telling us how it was last spring when it all began?

General Follow-up Questions

- Can you tell us more?
- What do you mean?
- How did you feel then?
- What were your thoughts at that time?
- What did you do?
- Can you give an example?

Areas of Inquiry

How have the demands on healthcare been managed?
Conditions/opportunities/vulnerabilities:

- Organization, working conditions, resources, management/governance
- Equipment, materials, premises, and resources
- Employee engagement, knowledge/competence, support, working methods, collaboration
- Information and communication
- Perceptions of patient safety, patient encounters, and contact with relatives
- Personal feelings and reflections.

In-depth Questions/Probes

- What did you do? Who contributed?
- How did it go? What was difficult? How did you succeed? Why? What were the conditions?
- Have decisions been made and followed? Adaptations in care and decisions? Anticipation? How has the situation been controlled?
- Consequences/results (desired/unwanted)?
- How has patient safety been affected?
- How has the work environment been affected?
- Any thoughts on ethics? Dilemmas?
- What weaknesses and strengths have influenced the changes?
- Learning over time? What happened next?

Personal Handling

- How have you been personally affected?
  - Work situation?
  - Ethical dilemmas? Existential thoughts?
  - What have you done to cope with the situation?
  - What has helped? What has made it more difficult? Consequences for you?

Current Situation - Recovery

- How is it now?
- How has the return to the "new normal" gone?
- What adaptations are being made? Preparations for a "second wave" / future crises?

Lessons Learned

- Ideas for the future?
- Success factors to carry with us?
- Anything else you wish to share?

Documents

- Are there any documents, such as procedures, guidelines, and decision logs that can help us understand what you have shared?

Thank You

- Contact details for occupational health services will be provided after the interview.
- Please feel free to contact us if you think of anything more you would like to share or if you have any questions.
