# Supplementary material for: Struggling to cope in an unknown realm: a qualitative study of moral resilience in frontline healthcare professionals in Swedish hospital wards during a pandemic crisis
Source: BMJ Open. 2026 Mar 23;16(3):e104065. doi: 10.1136/bmjopen-2025-104065 (PMC13034357; doi:10.1136/bmjopen-2025-104065)
Supplement: Supplementary file 2 [file bmjopen-2025-104065.reviewer_comments.pdf]

PEER REVIEW HISTORY

BMJ Open publishes all reviews undertaken for accepted manuscripts. Reviewers are asked to complete a checklist review form and are provided with free text boxes to elaborate on their assessment. These free text comments are reproduced below.

ARTICLE DETAILS

Title (Provisional)

Struggling to cope in an unknown realm: a qualitative study of moral resilience in frontline healthcare professionals in Swedish hospital wards during a pandemic crisis

Authors

Nordgren, Lena; Lohela Karlsson, Malin; Condén Mellgren, Emelie; Göras, Camilla; Bjurling-Sjöberg, Petronella

---

VERSION 1 - REVIEW

**Reviewer**	**1**
**Name**	**Rushton, Cynda**
**Affiliation**	**Johns Hopkins University, Berman Institute**
**Date**	**09-Aug-2025**
**COI**	**None**

This is a well designed and analyzed qualitative study of moral resilience among hospital personnel during early COVID-10 pandemic. Further work is needed to synthesize contemporary MR scholarship especially based on Rushton's conceptual model. Be careful not to go beyond your data in the discussion--see comments. Overall has the potential to be an important contribution to the evolving field of moral resilience.

**** **The reviewer provided a marked copy with additional comments. Please contact the publisher for full details.** ****

**Reviewer**	**2**
**Name**	**Newham, Roger**
**Affiliation**	**Buckinghamshire New University, Health and Social Care**
**Date**	**21-Oct-2025**
**COI**	**None**

Thank you for this.

A few minor comments.

The citation of the definition of moral resilience by Rushton is the wrong paper. You are using the updated definition in the 2018 book not the referenced 2016 paper.

A lot of space is given to the results. It would be helpful if an account of the type of theory produced is explained as it is grounded theory being used. This I think would add to the conceptual model which as such is quite general.

**Reviewer**	**3**
**Name**	**Monica Molinaro**
**Affiliation**	**McGill University**
**Date**	**10-Nov-2025**
**COI**	**None**

Thank you to the authors for their work on a very interesting and timely manuscript. My comments below are about how your interpretations could be bolstered and better supported, as well as adding strength to your methodology. I hope my comments are helpful.

I would recommend that any information relation to the number of participants recruited and the demographic characteristics of the participants (including Table 1) should be included in the results, and not the methods section of the manuscript. These elements are technically results and thus should be placed in that section. The sampling and participants section of the methods can simply be in regard to those who the team was hoping or planning to recruit.

I am wondering if the authors could provide more detail as to why they collected written narratives for a grounded theory study, specifically commenting on the methodological alignment between written narratives and grounded theory methodology. While I completely understand ease of participation for participants, written stories are not commonly used with grounded theory approaches.

I am also wondering if the authors can comment on why the study-specific guide needed to be reviewed and evaluated for its face validity as well.

Results
As a general note throughout the results section, there are some instances in which the descriptions made, and then the quotes used to illustrate those descriptions, are not aligned. By this I mean that the description is talking about something completely different than what the quote illustrates. I would suggest to the authors double check which quotes they are using from participants and why. As an example, in the Conflicting Loyalties section, I’m not sure the example quote that has been provided is the best example for this particular section, especially given that much of the description made afterward is about workplace conditions and the ability to be able to do work in relation to organizational priorities. Could another quote be used to illustrate this point more clearly? As another example, in the Development and Learning subsection, the quote from the infectious disease physician does not necessarily highlight how that person is ready to face challenges in the future – just that they learned a

lot from the experience. My suggestion would be to either find a different quote that is more illustrative of this point, or perhaps to reorganize some of the descriptions provided to be more aligned with what participants are saying. Or, if the analysis is inferring that this is what participants are narrating, then being more explicit about that interpretation would be helpful.

Another general note I have about the results section is that while moral resilience is described by the authors throughout, the connection between what participants were saying and how what they said is suggestive of moral resilience is not clear. Because the results read as more descriptive rather than interpretive, the reader has to do a lot of interpretive work to understand how specifically moral resilience comes into play. For the authors, I would suggest being more explicit in making these connections. Writing interpretive work is really tricky, and so something that has helped me immensely is writing as if I were to hold the reader by the hand and guide them through how the team came to understand that these quotes are illustrative of moral resilience. "By stating …., the participant suggests that…", or "when reading …., we came to understand that the participant was not only speaking about …, but also inferring ….". These kinds of statements help guide the reader through your thinking, which then also makes the connection between the quotes and moral resilience clearer as well.
As well, I would also note to the authors that at times the difference between resilience and moral resilience is not entirely clear. There are some instances in the manuscript, included in the discussion, where it seems as though resilience (and not moral resilience) is being discussed. To help with this, the points in your discussion about what moral resilience looks like, and how it can be fostered, are well done. I would recommend moving some of these points into your results section to explicitly highlight to the reader how your participants are engaging with moral resilience. From there, the discussion section can be used to draw attention to broader systemic issues, policy issues, institutional issues etc. that have contributed to moral distress and the need to moral resilience, and how moral resilience can possibly be fostered. The discussion section can also be used to highlight what other health care providers have experienced during the pandemic.

VERSION 1 - AUTHOR RESPONSE

Reviewer # 1 (Dr. Cynda Rushton, Johns Hopkins University)	
*This is a well designed and analyzed qualitative study of moral resilience among hospital personnel during early COVID-10 pandemic. Further work is needed to synthesize contemporary MR scholarship especially based on Rushton's conceptual model. Be careful not to go beyond your data in the discussion--see comments. Overall has the potential to be an important contribution to the evolving field of moral resilience.	Thank you for your encouraging words and for the thoughtful and constructive comments.
Method<br>P 14, line 1-2: "The majority of participants were female (85%), and over half were under 50 years old (63%)."<br>*How do these demographic compare with the study population--representative?	Thank you for this thoughtful comment.<br>• We have clarified the representativeness of our sample by specifying that the demographic distribution aligns with available statistics for the regional workforce from which the participants were recruited. The sentence in the Results section now reads:<br>"In line with demographics of the

	study population, the majority of participants..." (pp 11-12).
Results<br>P 19, line 14-15: "Work demands and resources /…/This heightened sense of fellowship reinforced moral resilience and facilitated their coping with the situation"<br>*This theme appears to be aligned with Rushton's relational integrity.	Thank you for highlighting this alignment; we fully agree. Based on this, we have made the following revisions:<br>• The subcategory has been renamed to *'Governance and interpersonal dynamics'* to more accurately reflect its content. Corresponding changes have been made in Table 2 and throughout the manuscript where the subcategory is referenced.<br>• In the Discussion section, we now explicitly acknowledge the alignment between these findings and Rushton's conceptualization of relational integrity.
P 25, line 18-19: "Safeguarding oneself to cope /…/ Others actively reduced their responsibilities to better manage stress and workload"<br>*This is an illustration of Rushton's self stewardship.	Thank you for this insightful comment. We agree with your interpretation, and we have made revisions accordingly.<br>• In the Results section, we clarified the example by adding: "…for example, by taking on tasks that did not require extensive decision-making*." (p. 25).*<br>• In the Discussion, we now explicitly link these self-protective strategies to Rushton's concept of self-stewardship and describe how they contribute to sustaining clinicians' well-being and moral integrity. (p. 32).
P 26, line 1-2: "To protect their well-being, some informants consciously framed themselves mentally to prevent excessive emotional impact from empathy."<br>*Unclear what this means--framed themselves mentally--do you mean compartmentalized?<br>*Do you mean to avoid empathic distress?	Thank you for highlighting this lack of clarity and for your helpful suggestions.<br>• We have revised the sentence accordingly. It now reads: "To preserve their well-being, some informants also described compartmentalizing their emotions to regulate empathy and mitigate excessive emotional strain. " (p. 26).
P 26, line 18-19: "However, this strategy seemed to contribute more to increased frustration than the intended relief."<br>*Important point!	Indeed, thank you!

P 28, line 5-6: "This indicates that moral resilience was not fully achieved, and the challenges were not navigated in a sustainable way"<br><br>*Isn't it also possible that people had varying degrees of moral resilience depending on the circumstances and that MR is not an end point but a state that is not static or immutable?<br><br>*It might be also be true that their capacities to address the ethical challenges were exceeded--not because they were deficient but rather that they were so intense that there was a threshold beyond which harm would occur. Or that their distress and exhaustion was caused by more than the ethical challenges alone.	Thank you for highlighting that our wording could be misinterpreted and unintentionally place responsibility on individuals.<br><br>• We agree with your proposed explanations and have revised the sentence accordingly. It now reads: "At times, the intensity of the moral challenges appeared to exceed their capacity to respond sustainably …" (p. 28).<br><br>• Additionally, we have adjusted the tense in several parts of the discussion to better anchor our interpretations in the data.
Discussion<br><br>P 30 line 9: "The model reveals a complex and dynamic iterative process, where components at both the individual and system levels are inevitably interrelated"<br><br>*This is consistent with Rushton's conceptualization of moral resilience as synergistic with an ethical practice environment. See Rushton (2024) Moral Resilience: Transforming Moral Suffering in Healthcare.	Thank you for this helpful suggestion.<br><br>• The discussion has been revised to clarify the relationship between individual- and system-level components in the model, with reference to prior conceptual work on moral resilience (p. 30).
P 31, line 1-2: "These strategies, characterized by team collaboration and readiness to intervene when necessary, were often perceived as more successful"<br><br>*This aligns with a core element of MR as described by Rushton as relational integrity.	Thank you for this valuable observation.<br><br>• We have revised the discussion to explicitly acknowledge that these team-based and collaborative strategies align with Rushton's concept of relational integrity. The text now reads: "Strategies characterized by team collaboration and readiness to intervene when needed were often perceived as more successful. Such collective approaches reflect what Rushton terms *relational integrity*, that is, acting in ways that uphold both one's own and others' moral commitments within the clinical environment". (p. 32).
P 31, line 6-7: "Additionally, group-based strategies, including support, are also crucial, with	Thank you for this helpful suggestion.

communication serving as a strategy at both the individual and group levels."<br><br>*Please review strategies as outlined by Rushton and others that support integrity and moral resilience. See reference #37 (Rushton CH. Transforming Moral Suffering by Cultivating Moral Resilience and Ethical Practice. American Journal of Critical Care. 2023, 32, 238-48. DOI: 20 10.4037/ajcc2023207.)	• We have revised the discussion to explicitly integrate insights from Rushton's work on strategies that support integrity and moral resilience. The text now acknowledges how the relational strategies identified in our data align with peer support, psychologically safe communication, and ethical support practices described by Rushton (2023) (p. 32).
P 31, line 8-9 "Insufficient capacity for moral resilience led to moral distress, and in certain cases, even moral injury"<br><br>*How are you distinguishing moral distress from moral injury---what data supports this claim?<br><br>P 31, line 9-10 "This was revealed to impair occupational wellbeing, with sometimes long-term consequences, affecting both the individual and the healthcare system at multiple levels."<br><br>*Please be more specific--include examples from your data.	Thank you for these important comments. We have clarified how we distinguish moral distress from moral injury and revised the text accordingly.<br><br>• The discussion now specifies that some participants described a more corrosive form of distress that imperiled their sense of integrity, consistent with definitions of moral injury (p. 32).<br><br>• We have also added concrete examples from the data, such as long-term sick leave and intentions to leave the profession, to illustrate the long-term consequences (p. 33).<br><br>• In the Results section, we have similarly clarified how emotional internalization sometimes escalated from moral distress toward moral injury (p. 27).
P 31, line 11-12: "Conversely, an adequate capacity for moral resilience was observed to facilitate personal development and growth in some individuals,…"<br><br>*How are you defining adequate?	Thank you for pointing out that the wording was unclear.<br><br>• We have revised the sentence to avoid implying a defined threshold of "adequate" capacity. The revised version now describes that when participants had the resources and support needed to navigate the moral challenges, this was sometimes associated with personal development and growth. We believe this phrasing more accurately reflects our empirical data and removes any unintended normative interpretation (p. 33).

P 31, line 14-15: "This confirms the conclusions of previous studies that certain individuals possess protective factors buffering them against moral distress [30, 31]."<br><br>*Please review studies included in reference 37 (Rushton CH. Transforming Moral Suffering by Cultivating Moral Resilience<br><br>19 and Ethical Practice. American Journal of Critical Care. 2023, 32, 238-48. DOI: 20 10.4037/ajcc2023207.).	Thank you for the helpful suggestion.<br><br>• We have reviewed the studies highlighted in Rushton (2023) and revised the manuscript accordingly. The paragraph now incorporates updated evidence demonstrating the protective role of moral resilience in relation to burnout, turnover intention, moral injury, and moral distress. (p. 33).
P 31, line 23: "All factors were manifested on a continuum, ranging from low to high levels…"<br><br>*How do you characterize high and low levels of MR? Did you measure?	Thank you for pointing out that our wording might be interpreted as implying quantitative measurement.<br><br>• We have revised the sentence to clarify that the continuum we refer to is an interpretive, qualitative description based on participants' accounts, not a measured construct. The revised text specifies that the factors appeared to vary along a continuum of more or less favorable conditions for moral resilience (p. 33).
P 32, line 8-11: "At an individual level, the following factors were identified as facilitators of moral resilience: a sense of meaningfulness, the ability to see the purpose of the role, contributions to teamwork, self-confidence, and feelings of empowerment. Loyalty, dedication, curiosity, interest, responsibility, positive traits, and humor were all linked to a sense of meaningfulness."<br><br>*How do these categories related to Rushton's MR capacities?	Thank you for this helpful comment.<br><br>• We have clarified how the categories relate to Rushton's conceptualization of moral resilience. A sentence has been added to explicitly link our findings to Rushton's key attributes of moral resilience. The revised text now reads:<br>*"These categories map well onto Rushton's [29] key attributes of moral resilience—integrity, moral efficacy, and self-regulation—suggesting that the individual facilitators identified in this study reflect core components of the construct. For instance, meaningfulness and purpose relate to moral integrity; teamwork contributions and empowerment reflect moral efficacy, while humor and positive traits may support emotional self-regulation."* (p. 34).
P 32, line 12-13: "Thus, the present findings align with the trait-state debate, resonating with the non-dualistic approach proposed by Kuldas et al [36]"	Thank you for this important observation.<br><br>• We have revised the wording to ensure we do not overstate what our

*How did you arrive at the conclusion that MR is a trait vs state.? This may go beyond your data.	data can support. We now describe our findings as resonating with, rather than demonstrating, the non-dualistic approach proposed by Kuldas et al., and we clarify why the interplay of factors observed in our data aligns with this perspective (p. 34).
P 33, line 1-2: … considerations are relevant from an interdisciplinary perspective and across various healthcare professions."<br>*This is reflected in later work to validate a scale to measure MR and it's use in interprofessional samples:<br>Rushton, C. H., Hanson, G. C., Boyce, D., Holtz, H., Nelson, K. E., Spilg, E. G., & Robillard, R. (2023). Reliability and validity of the revised Rushton Moral Resilience Scale-16 for healthcare workers. Journal of advanced nursing, 80(3), 1177–1187. https://doi.org/10.1111/jan.15873<br>Spilg, E., Rushton, C. Phillips, J., Kendzerska, T., Saad, M.,Gifford, W., Gautam, M., Bhatla, R., Edwards, J.,Quilty, L., Leveille, C, Robillar d , R. (2022) The new frontline: Exploring the links between moral distress, moral resilience, and mental health in healthcare workers during the COVID-19 pandemic, BMC Psychiatry. 2022) 22(19), 1-12. https://doi.org/10.1186/s12888-021-03637-w	Thank you for this important clarification.<br>• We have revised the discussion to clarify that moral resilience is relevant across healthcare professions and not limited to nursing. While Rushton's early work primarily focused on nurses, the revised text now acknowledges that subsequent research has extended the concept to interprofessional healthcare contexts. We therefore frame our findings in relation to this broader, interdisciplinary body of work, emphasizing that the considerations discussed are applicable across different healthcare professions (p. 35).
P 33, line 3-4: "Group-level factors, such as teamwork and collaboration, support from colleagues, solidarity, and diversity in experiences were also linked to moral resilience"<br>*Please review:<br>Rushton, C.H. Manbauman, C. (2023) Web of mutuality: Relational integrity in critical care nursing. AACN Advanced Critical Care.34(4), 381-390	*Thank you for this suggestion. We have reviewed Rushton & Manbauman (2023), and how the core concept of 'relational integrity' is elaborated in Rushton's book Moral Resilience (2023).*<br>• *Based on the review, we have added a reference to relational integrity in the discussion to clarify how our group-level findings align with this team-based dimension of the moral resilience framework (p. 35).*
P 33, line 16-18: "Notably, managers and physicians in the present study did not appear to face the same levels of uncertainty and lack of control as experienced by the nursing staff"<br>*Please review:<br>Rushton, C., Nelson, K, Bergman, A., Boyce, D., Jenkins, C., Swoboda, S., Hansen, G. (2024) Perceived	Thank you for this helpful suggestion. We have now reviewed Rushton et al. (2024). Although the study focuses on nurse leaders rather than interprofessional comparisons, it clearly demonstrates how organizational effectiveness, transparent decision-making, and psychological safety influence moral resilience and moral

Organizational Effectiveness during a Public Health Crisis and Moral Wellness among Nurse Leaders: A Cross-Sectional Study," Journal of Nursing Management, https://doi.org/10.1155/2024/6808266	injury. These insights support our interpretation that involvement in decision-making and access to clear communication pathways can reduce uncertainty and enhance a sense of control.<br>• We have now incorporated this reference and revised the text to reflect the relevance of leadership structures in shaping moral resilience (p. 36).
Strengths and limitations<br>P 36, line 1-2: "…conductive within the author group, which included researchers from diverse disciplines and with varied research backgrounds"<br>*It would be important to consider a mixed methods study to use quantitative data from a validated MR scale such as the RMRS-16 to triangulate with the qualitative analysis to verify conclusions.	Thank you for this valuable suggestion. We agree that integrating quantitative data from a validated moral resilience instrument could have enhanced methodological triangulation.<br>• We have added this point to the strengths and limitations section, noting that the use of a mixed-methods design could have strengthened the conclusions (p. 39).
Conclusions<br>P 36, line 7-9: "The results highlight that moral resilience is not solely dependent on individual traits but is also shaped by team dynamics, leadership, and organizational structures."<br>*This further confirms Rushton's premise that both individual and systemic strategies are needed to address the sources and consequences of moral adversity.	Thank you for this helpful comment. We fully agree that moral resilience arises from the interplay between individual capacities and systemic conditions.<br>• To clarify, we have clarified this point in the Conclusion by explicitly emphasizing that moral resilience is shaped by individual, relational, team-based, and organizational factors, consistent with Rushton's conceptualization (p. 39).
References<br>Ref. 11: "Rushton CH. Moral Resilience: A Capacity for Navigating Moral Distress in Critical Care. AACN Advanced Critical Care. 2016, 27, 111-9. DOI: 31 10.4037/aacnacc2016275. "<br>*A major source of scholarship on MR is missing: Rushton (ed) 2024 Moral Resilience: Transforming Moral Suffering in Healthcare, 2nd edition. OUP.	Thank you for noting this. We appreciate the importance of including the most recent and comprehensive source on moral resilience.<br>• We have now added Rushton's *Moral Resilience: Transforming Moral Suffering in Healthcare* (2nd edition, 2024) to the reference list and cited it where relevant throughout the manuscript.
Ref 41: "Rushton CH, Schoonover-Shoffner K, Kennedy MS. Executive Summary: Transforming Moral Distress into Moral Resilience in Nursing. The	Thank you for directing us to the updated edition. We have revised the section accordingly and now reference Rushton

American journal of nursing. 2017, 117, 52-6. DOI: 10.1097/01.Naj.0000512298.18641.31”<br><br>*Please review more recent data such as those included in Rushton (ed)(2024) Moral Resilience: Transforming Moral Suffering in Healthcare, 2nd edition.	(2024), which offers a more comprehensive and up-to-date synthesis of individual-level strategies for cultivating moral resilience. The 2024 edition expands on self-regulation practices such as mindfulness and highlights the central role of ethics education in strengthening moral awareness, ethical discernment, and moral agency.<br><br>• We have updated the manuscript to reflect these insights, and the previous citation has been replaced with the 2024 source.
Figure 1<br><br>“Conceptual model of the process of moral resilience among frontline healthcare professionals in a healthcare crisis”<br><br>*How does this model align or not with Rushton's conceptualization? Review Prior comments regarding 2nd edition of their book.(Rushton (ed) 2024 Moral Resilience: Transforming Moral Suffering in Healthcare, 2nd edition. OUP.)	Thank you for this important comment. Our conceptual model is empirically derived from frontline healthcare professionals’ experiences during a pandemic crisis and therefore reflects their situated accounts of navigating moral challenges. As such, it was not constructed to mirror any specific pre-existing theoretical framework.<br><br>Following the reviewer’s suggestion, we have reviewed Rushton’s updated conceptualization of moral resilience (Rushton, 2024). We find that several aspects of our empirically grounded model resonate with key components of Rushton’s framework—for example:<br>• its dynamic and process-oriented nature,<br>• the interplay between individual capacities and relational/organizational conditions, and<br>• the central role of responding to moral adversity.<br><br>At the same time, our model includes context-specific categories that emerged inductively from the data, reflecting the unique challenges faced in general hospital wards during the pandemic.<br><br>• To make this alignment and distinction explicit, we have added a brief statement in the Discussion section clarifying where our findings converge with Rushton’s conceptualization and where they extend or differ from it (p. 30).

Reviewer # 2: (Dr. Roger Newham)	
Thank you for this. A few minor comments.<br>* The citation of the definition of moral resilience by Rushton is the wrong paper. You are using the updated definition in the 2018 book not the referenced 2016 paper.	Thank you for this important clarification.<br>• After reviewing suggested references, we have corrected the citation in order to correctly reflect the definition in the 2016 paper.
* A lot of space is given to the results. It would be helpful if an account of the type of theory produced is explained as it is grounded theory being used. This I think would add to the conceptual model which as such is quite general.	Thank you for demonstrating that we have been unclear in this matter and giving us the opportunity to provide clarification. We have made amendments accordingly, both in the Methods, Results and Discussion.<br>• In the Analysis section, we have added the following:<br>"In line with grounded theory methodology, theorizing was an interpretative process. This process could aim for different levels of abstraction, ranging from conceptual ordering to theory creation [25]. In this study, we aimed at conceptual ordering, and created a conceptual model based on the theoretical scheme. " (p. 10).<br>• In the results, when explaining the structure of the section, we have added the following:<br>"The main text first explains the conceptual model, including the core category, context and interrelations between the categories." (p. 12).<br>• Additionally, we have changed the subheading in the beginning of the Results and revised the text about the conceptual model to facilitate understanding. This subheading now reads: "The process of moral resilience in the context of a novel pandemic crisis – a conceptual model". (p. 13).<br>• We have added a subheading for the core category. The new subheading reads:<br>"A struggle to cope in an unknown realm" (p. 14).

	• In the category subheading 'Exposure to moral challenges' we have added a sentence to further clarify the process. This reads as follows:<br>… These situations had the potential to cause moral distress and tested the healthcare professionals' ability to maintain their integrity and uphold ethical standards, that is, their moral resilience. How they responded to the moral challenges are further explained below, in the category 'Various strategies'. (p. 15).<br><br>• In the Methodological strength and limitations section, we have added the following:<br>"Grounded theory research can be directed towards different levels of abstraction, ranging from conceptual ordering to theory creation [25]. This study aimed at conceptual ordering. Nevertheless, the developed conceptual model has explanatory power regarding the substantive area explored, suggesting that it may be regarded as a substantive theory." (p. 37).<br><br>• In the bullet points, we have added the following:<br>"This grounded theory study combined individual and system-level perspectives, resulting in a conceptual model of the process of moral resilience, including its dimensions, affecting factors, and consequences, which can contribute to the theoretical development within the field." (p. 5).
Reviewer # 3: (Monica Molinaro, McGill University)	
*Thank you to the authors for their work on a very interesting and timely manuscript. My comments below are about how your interpretations could be bolstered and better supported, as well as adding strength to your methodology. I hope my comments are helpful.	Thank you for the review and the encouraging words. We appreciate your suggestions, which we have addressed as outlined point-by-point below.
Method	Thank you for the suggestion.

*I would recommend that any information relation to the number of participants recruited and the demographic characteristics of the participants (including Table 1) should be included in the results, and not the methods section of the manuscript. These elements are technically results and thus should be placed in that section. The sampling and participants section of the methods can simply be in regard to those who the team was hoping or planning to recruit.	• We have moved the information regarding number of participants recruited and the demographic characteristics of the participants (including Table 1) from the Method to the Results. We also shifted upward the existing related text in the Results (p. 11-12).
*I am wondering if the authors could provide more detail as to why they collected written narratives for a grounded theory study, specifically commenting on the methodological alignment between written narratives and grounded theory methodology. While I completely understand ease of participation for participants, written stories are not commonly used with grounded theory approaches.	Thank you for highlighting this point. We agree that our rationale and methodological justification for including written narratives required clarification. We have now addressed this both in the Methods and in the Discussion.<br>• To clarify the rationale, the Data collection section now reads as follows: "At the time of data collection, healthcare professionals were still grappling with the strains of the pandemic. To capture a broad range of experiences without adding to participants' burden, minimally demanding and flexible data collection methods were employed...." (p. 9).<br>• **To strengthen the methodological alignment with grounded theory, we added some text in Methodological strengths and limitations section (p. 38).**
*I am also wondering if the authors can comment on why the study-specific guide needed to be reviewed and evaluated for its face validity as well.	Thank you for the question and the opportunity to clarify this point. The interview guide was originally developed within the larger ResCOV research project. As part of that broader development process, the guide underwent a face validity assessment to ensure that potential informants would clearly understand the intent of each question. Based on this feedback, we made minor linguistic adjustments prior to using the guide in the present study.<br><br>We acknowledge that the brief mention of this procedure in the submitted

	manuscript may create confusion rather than add methodological clarity.<br><br>• Because the full description of the guide development—including the face validity process—is already provided in the referenced study protocol, we have removed this sentence from the manuscript to avoid unnecessary redundancy.
Results<br>*As a general note throughout the results section, there are some instances in which the descriptions made, and then the quotes used to illustrate those descriptions, are not aligned. By this I mean that the description is talking about something completely different than what the quote illustrates. I would suggest to the authors double check which quotes they are using from participants and why.<br><br>*As an example, in the Conflicting Loyalties section, I'm not sure the example quote that has been provided is the best example for this particular section, especially given that much of the description made afterward is about workplace conditions and the ability to be able to do work in relation to organizational priorities. Could another quote be used to illustrate this point more clearly?<br><br>*As another example, in the Development and Learning subsection, the quote from the infectious disease physician does not necessarily highlight how that person is ready to face challenges in the future – just that they learned a lot from the experience. My suggestion would be to either find a different quote that is more illustrative of this point, or perhaps to reorganize some of the descriptions provided to be more aligned with what participants are saying. Or, if the analysis is inferring that this is what participants are narrating, then being more explicit about that interpretation would be helpful.	Thank you for drawing our attention to these important issues regarding alignment between descriptions and illustrative quotations. We fully agree that the original version contained instances where the connection between the analytic text and the selected quotes was not sufficiently clear.<br><br>In response, we have undertaken the following revisions:<br><br>• Systematically reviewed all quotes in the Results section to ensure that each quotation clearly supports the analytic point it is intended to illustrate.<br>• Revised and clarified several analytic descriptions to improve contextualization and strengthen the connection between data and interpretation.<br>• Repositioned some quotations to sections where they more accurately exemplify the themes and subcategories.<br>• Clarified interpretive steps where the analysis moved beyond the literal wording of the quotation, making this explicit in the surrounding text.<br>These revisions have been made throughout the Results section. Specific changes include adjustments within *Conflicting loyalties, Ethical challenges in clinical practice, Governance and interpersonal dynamics (previously Work demands and resources), Meaningfulness and energy, Engagement with management and organizational issues, Autonomous patient care practices, Safeguarding oneself to cope,* and

	Development and learning. In the latter case, the quote from the infectious disease physician has been relocated, and the analytic narrative now concludes with two quotations that more clearly illustrate learning and preparedness for future challenges.<br><br>We believe these revisions have substantially improved the coherence, transparency, and analytic clarity of the Results section.<br><br>All revisions described above are clearly marked in the manuscript using the journal's recommended method for indicating changes (track changes).
*Another general note I have about the results section is that while moral resilience is described by the authors throughout, the connection between what participants were saying and how what they said is suggestive of moral resilience is not clear. Because the results read as more descriptive rather than interpretive, the reader has to do a lot of interpretive work to understand how specifically moral resilience comes into play. For the authors, I would suggest being more explicit in making these connections. Writing interpretive work is really tricky, and so something that has helped me immensely is writing as if I were to hold the reader by the hand and guide them through how the team came to understand that these quotes are illustrative of moral resilience. "By stating …., the participant suggests that…", or "when reading …., we came to understand that the participant was not only speaking about …, but also inferring ….". These kinds of statements help guide the reader through your thinking, which then also makes the connection between the quotes and moral resilience clearer as well.	Thank you very much for this thoughtful and constructive guidance. We agree that the interpretive links between participants' accounts and the concept of moral resilience needed to be made more explicit. Following your suggestion, we have revised the Results section to better guide the reader through our analytical reasoning.<br><br>• We have clarified the presentation of the conceptual model (as described in response to your next comment), making the interpretive logic more transparent (p.13).<br>• Across the Results section, we have strengthened interpretive transitions between the data and the emerging concepts. Specifically, we have added brief interpretive phrases—e.g., *"which appeared to…", "which we interpreted as…", "indicating that…", "this seemed to reflect their moral resilience…"*—to explicitly articulate how participants' descriptions were understood analytically.<br><br>These additions aim to more clearly demonstrate how the participants' accounts informed our interpretation of moral resilience, in line with grounded theory methodology. All revisions are marked in the manuscript.

Result & Discussion<br>As well, I would also note to the authors that at times the difference between resilience and moral resilience is not entirely clear. There are some instances in the manuscript, included in the discussion, where it seems as though resilience (and not moral resilience) is being discussed. To help with this, the points in your discussion about what moral resilience looks like, and how it can be fostered, are well done. I would recommend moving some of these points into your results section to explicitly highlight to the reader how your participants are engaging with moral resilience.	Thank you for this important observation. We agree that the distinction between *resilience* and *moral resilience* was not always sufficiently clear, and we have revised the manuscript accordingly.<br><br>• Clarification in the Methods section: We have added a brief explanation situating the study within the broader ResCOV project, which examined resilience from multiple perspectives. This clarifies why both resilience and moral resilience appear in the manuscript, and how our study specifically focuses on moral resilience.<br>• Strengthening interpretive clarity in the Results:<br>In the section introducing the conceptual model, we have revised the wording to explicitly emphasize that the model describes a *moral resilience process*. We now more clearly articulate how the core category, main categories, and subcategories relate specifically to moral resilience, and how varying *levels of moral resilience* shaped participants' experiences. These revisions help distinguish moral resilience from general resilience throughout the Results. (p.13)<br>• Precision in terminology:<br>In instances where participants were referring to resilience at a system or organizational level—rather than moral resilience—we now explicitly use the term *organizational resilience* to avoid conceptual ambiguity.<br>These revisions aim to provide a clearer conceptual boundary between resilience and moral resilience and to ensure that the reader can confidently follow how participants' accounts relate to the moral resilience process. All changes are marked in the manuscript.
From there, the discussion section can be used to draw attention to broader systemic issues, policy issues, institutional issues etc. that have contributed	Thank you for this suggestion. We have revised the Discussion to more explicitly address how systemic, organizational, and policy-level factors contribute to moral distress and shape the need for moral

to moral distress and the need to moral resilience, and how moral resilience can possibly be fostered.	resilience. The revised text highlights structural drivers such as resource scarcity, redeployment, information asymmetries, and institutional support, and links these conditions to the fostering of moral resilience at organizational as well as individual and relational levels (p. 31).
The discussion section can also be used to highlight what other health care providers have experienced during the pandemic.	Thank you for this helpful suggestion. In response, we have expanded the discussion to explicitly situate our findings in relation to experiences reported by healthcare professionals in other settings during the COVID-19 pandemic.<br>• We now highlight that several challenges described by our participants resemble issues widely observed across healthcare, thereby underscoring that the moral adversities and coping strategies identified in our study align with broader patterns across professions (p. 35).

VERSION 2 - REVIEW

**Reviewer**	**1**
**Name**	**Rushton, Cynda**
**Affiliation**	**Johns Hopkins University, Berman Institute**
**Date**	**23-Feb-2026**
**COI**	

Well-done revisions! A few tweaks but overall comprehensive.

**** **The reviewer provided a marked copy with additional comments. Please contact the publisher for full details.** ****

VERSION 2 - AUTHOR RESPONSE

Reviewer # 1 (Dr. Cynda Rushton, Johns Hopkins University)	
Reviewer: Well-done revisions! A few tweaks but overall comprehensive.	Thank you! We appreciate your effort as a reviewer.
P 31: "The model aligns with key elements of Rushton's [29] conceptualization of moral resilience— particularly its dynamic, process-oriented character and the interplay between individual capacities and relational or organizational conditions."<br><br>Reviewer: Please elaborate on these elements	Thank you for the suggestion.<br><br>We have elaborated on these elements and made applicable adjustments of related sentences. This part of the manuscript now reads:<br>"The findings contribute to existing knowledge by offering empirically grounded insights into multiple dimensions of moral resilience, its consequences, and influencing factors. The model aligns with the key elements of moral resilience highlighted by Rushton [29]—personal and relational integrity as the ethical anchor, self-regulation and self-awareness supporting the individuals response to moral stressors, and moral efficacy referring to the confidence in one's capacity to act in accordance with moral commitments despite adversity. At the same time, the model incorporates context-specific categories grounded in frontline professionals' experiences during the COVID-19 pandemic, thereby extending and nuancing existing theoretical work. Particularly the dynamic, process-oriented character of the phenomenon and the interplay between individual capacities and relational or organizational conditions are displayed." (P 30-31)
P 32: "Moral resilience is consistently associated with lower distress and better mental health across professions, whereas exposures such as redeployment and end-of-life constraints are linked to adverse outcomes [10]."<br><br>Reviewer: Consider adding additional references to support this conclusion.	We agree with your suggestion and have added relevant references to strengthen this conclusion (P 31).
P 33, line 4: "The strategies ranged from passive to proactive responses and operated across different system levels. For example, the strategy of *suffering in silence* eroded individuals´ integrity over time.	This is a relevant question. While one might hope that the motivation also served the patients' best interests, in this

Some individualistic strategies—such as raising concerns for personal benefit— were more proactive but not consistently effective."<br><br>Reviewer: Was this [personal benefit] only for personal benefit or was it also related to benefiting the patient?	case it primarily pertained to one's own benefit rather than the patients'.
P 34: In some cases, these consequences resulted in long-term sick leave or intentions to leave the profession, affecting both individuals and the healthcare system.<br><br>Reviewer: Link these findings to research findings that indicate that lower MR is associated with burnout and intent to leave.	We agree with your suggestion and have added relevant references to link these findings to previous publications (P 33).
P 34: "It is also noteworthy that individuals in the same environment may exhibit varying levels of moral resilience [32]."<br><br>Reviewer: This is an important point!	Indeed, thank you!
P 37: "At the **individual level**, relevant components include stress-management and self-regulation practices—such as mindfulness or meditation—to support emotional regulation, as well as ethics education aimed at strengthening ethical discernment, moral awareness, and the ability to translate values into action when facing morally complex situations [29]."<br><br>Reviewer: It also encompasses a commitment to know oneself, to choose actions that are wholesome and healthy and to commit to taking action to support one's own wellbeing while compassionately honoring one's limitations. through self-stewardship.	Thank you for highlighting this matter. We agree and have added a sentence about this. (P 37)
P 38: "At the **organizational level**, measures may include… "<br><br>Reviewer: At the organizational level it also includes having clear policies for resource allocation, transparent processes for reporting safety or ethically challenging events, and having dedicated resources to support staff to address ethical challenges.	Thank you for this helpful suggestion. We agree and have added a sentence accordingly. (P 37)
